# Supplementary material for: Impact of short-term change of adiposity on risk of high blood pressure in children: Results from a follow-up study in China
Source: PLoS One. 2021 Sep 10;16(9):e0257144. doi: 10.1371/journal.pone.0257144 (PMC8432865; doi:10.1371/journal.pone.0257144)
Supplement: S1 Table — (DOCX) [file pone.0257144.s001.docx]

| **S1 Table. Body Mass Index Reference Norm for Screening Overweight and Obesity Among Chinese children aged 6-18 years (kg/m^2^)** | | | | | |
| --- | --- | --- | --- | --- | --- |
| **Age** | **Boys** | |  | **Girls** | |
| **(years)** | **Overweight** | **Obesity** |  | **Overweight** | **Obesity** |
| 6.0～ | 16.4 | 17.7 |  | 16.2 | 17.5 |
| 6.5～ | 16.7 | 18.1 |  | 16.5 | 18.0 |
| 7.0～ | 17.0 | 18.7 |  | 16.8 | 18.5 |
| 7.5～ | 17.4 | 19.2 |  | 17.2 | 19.0 |
| 8.0～ | 17.8 | 19.7 |  | 17.6 | 19.4 |
| 8.5～ | 18.1 | 20.3 |  | 18.1 | 19.9 |
| 9.0～ | 18.5 | 20.8 |  | 18.5 | 20.4 |
| 9.5～ | 18.9 | 21.4 |  | 19.0 | 21.0 |
| 10.0～ | 19.2 | 21.9 |  | 19.5 | 21.5 |
| 10.5～ | 19.6 | 22.5 |  | 20.0 | 22.1 |
| 11.0～ | 19.9 | 23.0 |  | 20.5 | 22.7 |
| 11.5～ | 20.3 | 23.6 |  | 21.1 | 23.3 |
| 12.0～ | 20.7 | 24.1 |  | 21.5 | 23.9 |
| 12.5～ | 21.0 | 24.7 |  | 21.9 | 24.5 |
| 13.0～ | 21.4 | 25.2 |  | 22.2 | 25.0 |
| 13.5～ | 21.9 | 25.7 |  | 22.6 | 25.6 |
| 14.0～ | 22.3 | 26.1 |  | 22.8 | 25.9 |
| 14.5～ | 22.6 | 26.4 |  | 23.0 | 26.3 |
| 15.0～ | 22.9 | 26.6 |  | 23.2 | 26.6 |
| 15.5～ | 23.1 | 26.9 |  | 23.4 | 26.9 |
| 16.0～ | 23.3 | 27.1 |  | 23.6 | 27.1 |
| 16.5～ | 23.5 | 27.4 |  | 23.7 | 27.4 |
| 17.0～ | 23.7 | 27.6 |  | 23.8 | 27.6 |
| 17.5～ | 23.8 | 27.8 |  | 23.9 | 27.8 |
| 18.0～ | 24.0 | 28.0 |  | 24.0 | 28.0 |

Notes: National Health Commission of the People’s Republic of China: **Screening for overweight and obesity amng school-age children and adolescents**. WS/T 586—2018; 2018.
