# Supplementary material for: Impact of short-term change of adiposity on risk of high blood pressure in children: Results from a follow-up study in China
Source: PLoS One. 2021 Sep 10;16(9):e0257144. doi: 10.1371/journal.pone.0257144 (PMC8432865; doi:10.1371/journal.pone.0257144)
Supplement: S2 Table — (DOCX) [file pone.0257144.s002.docx]

| **S2 Table. The prevalence of obesity in children based on two international definitions of child obesity** | | | | | | | | | |
| --- | --- | --- | --- | --- | --- | --- | --- | --- | --- |
| Variables | | Group | Boys | | Girls | | Total | | *P* value |
|  |  |  | (n=14542) | | (n=13746) | | (N=28288) | |  |
|  |  |  | N/Mean | %/SD | N/Mean | %/SD | N/Mean | %/SD | |
| ***Prevalence at the Baseline survey*** | | | | | | | | | |
| ITOF standard^a^ | General obesity | no | 13325 | 91.60% | 13249 | 96.40% | 26574 | 93.90% | <0.001 |
|  |  | yes | 1217 | 8.40% | 497 | 3.60% | 1714 | 6.10% |  |
| WHO  standard^b^ | General obesity | no | 12385 | 85.20% | 13038 | 94.80% | 25423 | 89.90% | <0.001 |
|  |  | yes | 2157 | 14.80% | 708 | 5.20% | 2865 | 10.10% |  |
| ***Prevalence at the follow-up survey*** | | | | | | | | | |
| ITOF standard^a^ | General obesity | no | 13531 | 93.00% | 13327 | 97.00% | 26858 | 94.90% | <0.001 |
|  |  | yes | 1011 | 7.00% | 419 | 3.00% | 1430 | 5.10% |  |
| WHO  standard^b^ | General obesity | no | 12711 | 87.40% | 13096 | 95.30% | 25807 | 91.20% | <0.001 |
|  |  | yes | 1831 | 12.60% | 650 | 4.70% | 2481 | 8.80% |  |
| ***Change of obesity status from baseline to follow-up*** | | | | | | | | | |
| ITOF standard^a^ | General obesity status change^c^ | NN | 13240 | 91.00% | 13198 | 96.00% | 26438 | 93.50% | <0.001 |
|  |  | YN | 291 | 2.00% | 129 | 0.90% | 420 | 1.50% |  |
|  |  | NY | 85 | 0.60% | 51 | 0.40% | 136 | 0.50% |  |
|  |  | YY | 926 | 6.40% | 368 | 2.70% | 1294 | 4.60% |  |
| WHO  standard^b^ | General obesity status change^c^ | NN | 12246 | 84.20% | 12956 | 94.30% | 25202 | 89.10% | <0.001 |
|  |  | YN | 465 | 3.20% | 140 | 1.00% | 605 | 2.10% |  |
|  |  | NY | 139 | 1.00% | 82 | 0.60% | 221 | 0.80% |  |
|  |  | YY | 1692 | 11.60% | 568 | 4.10% | 2260 | 8.00% |  |
| a ITOF standard: the International Obesity Task Force (IOTF) standard. b WHO standard: the World Health Organization (WHO) standard. c NN: non-obese at baseline and non-obese at follow-up; NY: non-obese at baseline and obese at follow-up; YN: obese at baseline and non-obese at follow-up; YY: obese at baseline and obese at follow-up. | | | | | | | | | |
