# Supplementary material for: Impact of short-term change of adiposity on risk of high blood pressure in children: Results from a follow-up study in China
Source: PLoS One. 2021 Sep 10;16(9):e0257144. doi: 10.1371/journal.pone.0257144 (PMC8432865; doi:10.1371/journal.pone.0257144)
Supplement: S3 Table — (DOCX) [file pone.0257144.s003.docx]

| **S3 Table. Association between risk of HBP and different obesity status change with adjustment of other potential covariates** | | | | | |
| --- | --- | --- | --- | --- | --- |
| Obesity type | Group^a^ | Model 1^b^ | | Model 2^c^ | |
|  |  | OR (95%CI) | *P* | OR (95%CI) | *P* |
| General obesity | NN | 1(Ref.) |  |  |  |
|  | YN | 2.83(2.11~3.8) | <0.001 | 2.51(1.87~3.36) | <0.001 |
|  | NY | 3.45(2.05~5.82) | <0.001 | 3.19(1.88~5.42) | <0.001 |
|  | YY | 5.32(4.54~6.23) | <0.001 | 4.41(3.77~5.16) | <0.001 |
| Abdominal obesity | NN | 1(Ref.) |  |  |  |
|  | YN | 2.26(1.77~2.87) | <0.001 | 2.13(1.67~2.72) | <0.001 |
|  | NY | 3.43(2.37~4.97) | <0.001 | 3.32(2.28~4.83) | <0.001 |
|  | YY | 4.85(4.14~5.69) | <0.001 | 4.13(3.51~4.85) | <0.001 |
| ^a^NN: non-obese at baseline and non-obese at follow-up; NY: non-obese at baseline and obese at follow-up; YN: obese at baseline and non-obese at follow-up; YY: obese at baseline and obese at follow-up. ^b^Adjusted for age, gender, province, area, fruits consumption, vegetable consumption, sugar-sweetened beverage intake, physical activity and height change. ^c^Adjusted for age, gender, province, area, fruits consumption, vegetable consumption, sugar-sweetened beverage intake, physical activity and baseline HBP (high blood pressure) status. | | | | | |
