# Supplementary material for: Impact of short-term change of adiposity on risk of high blood pressure in children: Results from a follow-up study in China
Source: PLoS One. 2021 Sep 10;16(9):e0257144. doi: 10.1371/journal.pone.0257144 (PMC8432865; doi:10.1371/journal.pone.0257144)
Supplement: S4 Table — (DOCX) [file pone.0257144.s004.docx]

| **S4 Table. Association between risk of high blood pressure and different groups (ten equal groups) of BMI or WHtR change in the obese children** | | | | | | | |
| --- | --- | --- | --- | --- | --- | --- | --- |
| Variables | Percentile | | Range | Model 1 | | Model 2 | |
|  |  |  |  | OR (95%CI) | *P* | OR (95%CI) | *P* |
| BMI change (kg/m^2^) | ≤P_10_ | ≤ -1.43 | | 0.70(0.49~1.01) | 0.056 | 0.63(0.43~0.91) | 0.013 |
|  | P_10_~P_20_ | -1.43 ~ -0.88 | | 0.69(0.48~0.99) | 0.042 | 0.62(0.43~0.90) | 0.013 |
|  | P_20_~P_30_ | -0.88~ -0.51 | | 0.70(0.49~1.00) | 0.051 | 0.67(0.46~0.97) | 0.034 |
|  | P_30_~P_40_ | -0.51~ -0.21 | | 0.72(0.51~1.04) | 0.076 | 0.68(0.47~0.99) | 0.042 |
|  | P_40_~P_50_ | -0.21~ 0.07 | | 0.79(0.56~1.12) | 0.182 | 0.77(0.54~1.10) | 0.146 |
|  | P_50_~P_60_ | 0.07~0.31 | | 0.76(0.53~1.08) | 0.121 | 0.78(0.54~1.12) | 0.171 |
|  | P_60_~P_70_ | 0.31~0.59 | | 0.88(0.62~1.23) | 0.452 | 0.89(0.63~1.27) | 0.528 |
|  | P_70_~P_80_ | 0.59~0.93 | | 1.17(0.85~1.61) | 0.341 | 1.17(0.85~1.63) | 0.339 |
|  | P_80_~P_90_ | 0.93~1.47 | | 1(Ref.) |  | 1(Ref.) |  |
|  | >P_90_ | >1.47 | |  |  |  |  |
| WHtR change | ≤P_10_ | ≤ -0.063 | | 0.74(0.52~1.03) | 0.076 | 0.5(0.35~0.74) | <0.001 |
|  | P_10_~P_20_ | -0.063 ~ -0.042 | | 0.55(0.38~0.8) | 0.002 | 0.45(0.3~0.67) | <0.001 |
|  | P_20_~P_30_ | -0.042~ -0.032 | | 0.67(0.47~0.95) | 0.026 | 0.64(0.44~0.92) | 0.016 |
|  | P_30_~P_40_ | -0.032~ -0.023 | | 0.73(0.52~1.03) | 0.073 | 0.75(0.53~1.07) | 0.110 |
|  | P_40_~P_50_ | -0.023~ -0.015 | | 0.65(0.45~0.92) | 0.016 | 0.66(0.46~0.95) | 0.025 |
|  | P_50_~P_60_ | -0.015~ -0.008 | | 0.74(0.52~1.03) | 0.076 | 0.75(0.53~1.06) | 0.103 |
|  | P_60_~P_70_ | -0.008~ -0.001 | | 1.18(0.87~1.59) | 0.297 | 1.18(0.86~1.61) | 0.298 |
|  | P_70_~P_80_ | -0.001~ 0.008 | | 0.87(0.63~1.2) | 0.397 | 0.88(0.63~1.22) | 0.435 |
|  | P_80_~P_90_ | 0.008~ 0.020 | | 1(Ref.) |  | 1(Ref.) |  |
|  | >P_90_ | >0.020 | |  |  |  |  |
| Model 1 is the crude model. Model 2 is adjusted for age, gender, province, and area. BMI: body mass index. WHtR: waist-to-height ratio. | | | | | | | |
