# Supplementary material for: Impact of short-term change of adiposity on risk of high blood pressure in children: Results from a follow-up study in China
Source: PLoS One. 2021 Sep 10;16(9):e0257144. doi: 10.1371/journal.pone.0257144 (PMC8432865; doi:10.1371/journal.pone.0257144)
Supplement: S5 Table — (DOCX) [file pone.0257144.s005.docx]

| **S5 Table. Blood pressure level and risk of high blood pressure of different pattern of general obesity based on two international definitions of child obesity** | | | | | | | | | | |  |  |  |
| --- | --- | --- | --- | --- | --- | --- | --- | --- | --- | --- | --- | --- | --- |
| **Obesity definition** | | **Variables** | | ***General obesity status change^c^*** | | | | | | | | | ***P* value** |
|  |  |  |  | **NN** | | **YN** | | **NY** | | **YY** | | |  |
|  |  |  |  | **N/Mean** | **%/SD** | **N/Mean** | **%/SD** | **N/Mean** | **P value** | **N/Mean** | **%/SD** | |  |
| ITOF standard^a^ | | SBP_baseline_ | | 103.65 | 11.64 | 110.64 | 12.84 | 109.64 | 12.71 | 113.73 | 12.83 | | <0.001 |
|  |  | SBP_follow-up_ | | 103.84 | 10.95 | 110.76 | 12.01 | 112.31 | 11.71 | 115.51 | 12.56 | | <0.001 |
|  |  | SBP change | | 0.2 | 12.25 | 0.12 | 13.74 | 2.67 | 15.01 | 1.78 | 14.16 | | <0.001 |
|  |  | DBP_baseline_ | | 65.9 | 8.49 | 70.42 | 9.63 | 69.49 | 8.51 | 71.74 | 8.67 | | <0.001 |
|  |  | DBP_follow-up_ | | 65.32 | 8.1 | 69.85 | 9.32 | 69.31 | 8.5 | 72.12 | 8.49 | | <0.001 |
|  |  | DBP change | | -0.58 | 10.14 | -0.56 | 11.38 | -0.17 | 11.1 | 0.38 | 10.64 | | 0.011 |
|  |  | HBP_baseline_ | no | 24159 | 91.40% | 325 | 77.40% | 109 | 80.10% | 965 | 74.60% | | <0.001 |
|  |  |  | yes | 2279 | 8.60% | 95 | 22.60% | 27 | 19.90% | 329 | 25.40% | |  |
|  |  | HBP_follow-up_ | no | 25156 | 95.20% | 356 | 84.80% | 114 | 83.80% | 1010 | 78.10% | | <0.001 |
|  |  |  | yes | 1282 | 4.80% | 64 | 15.20% | 22 | 16.20% | 284 | 21.90% | |  |
| WHO standard^b^ | | SBP_baseline_ | | 103.42 | 11.58 | 107.2 | 12.2 | 107.9 | 12.7 | 112.26 | 12.59 | | <0.001 |
|  |  | SBP_follow-up_ | | 103.57 | 10.89 | 108.43 | 11.01 | 110.95 | 10.83 | 113.4 | 12.32 | | <0.001 |
|  |  | SBP change | | 0.16 | 12.18 | 1.23 | 13.64 | 3.06 | 14.15 | 1.14 | 13.91 | | <0.001 |
|  |  | DBP_baseline_ | | 65.77 | 8.46 | 68.09 | 8.83 | 68.63 | 9.37 | 70.89 | 8.86 | | <0.001 |
|  |  | DBP_follow-up_ | | 65.16 | 8.07 | 68.14 | 8.13 | 69.65 | 8.82 | 70.96 | 8.61 | | <0.001 |
|  |  | DBP change | | -0.61 | 10.08 | 0.05 | 10.68 | 1.02 | 12.2 | 0.07 | 10.97 | | 0.001 |
|  |  | HBP_baseline_ | no | 23132 | 91.80% | 515 | 85.10% | 181 | 81.90% | 1730 | 76.50% | | <0.001 |
|  |  |  | yes | 2070 | 8.20% | 90 | 14.90% | 40 | 18.10% | 530 | 23.50% | |  |
|  |  | HBP_follow-up_ | no | 24059 | 95.50% | 536 | 88.60% | 185 | 83.70% | 1856 | 82.10% | | <0.001 |
|  |  |  | yes | 1143 | 4.50% | 69 | 11.40% | 36 | 16.30% | 404 | 17.90% | |  |
| SBP: systolic blood pressure. DBP: diastolic blood pressure. HBP: high blood pressure.  a ITOF standard: the International Obesity Task Force (IOTF) standard.  b WHO standard: the World Health Organization (WHO) standard.  c NN: non-obese at baseline and non-obese at follow-up; NY: non-obese at baseline and obese at follow-up; YN: obese at baseline and non-obese at follow-up; YY: obese at baseline and obese at follow-up. | | | | | | | | | | | | | |
