# Supplementary material for: Impact of short-term change of adiposity on risk of high blood pressure in children: Results from a follow-up study in China
Source: PLoS One. 2021 Sep 10;16(9):e0257144. doi: 10.1371/journal.pone.0257144 (PMC8432865; doi:10.1371/journal.pone.0257144)
Supplement: S6 Table — (DOCX) [file pone.0257144.s006.docx]

| **S6 Table. Association between risk of high blood pressure and different general or abdominal obesity status change based on two international definitions of child obesity** | | | | | | | | | | |
| --- | --- | --- | --- | --- | --- | --- | --- | --- | --- | --- |
| Obesity definition | Obesity type | Group^a^ | Model 1 | | Model 2 | | Model 3 | | Model 4 | |
|  |  |  | OR(95%CI) | *P* | OR(95%CI) | *P* | OR(95%CI) | *P* | OR(95%CI) | *P* |
| ITOF standard^a^ | General obesity status change^c^ | NN | 1(Ref.) |  | 1(Ref.) |  | 1(Ref.) |  | 1(Ref.) |  |
|  |  | YN | 3.53(2.69,4.63) | <0.001 | 3.26(2.48,4.29) | <0.001 | 2.91(2.20,3.85) | <0.001 | 3.51(2.52,4.90) | <0.001 |
|  |  | NY | 3.79(2.39,6.00) | <0.001 | 3.67(2.31,5.82) | <0.001 | 4.19(2.61,6.73) | <0.001 | 3.98(2.21,7.15) | <0.001 |
|  |  | YY | 5.52(4.78,6.37) | <0.001 | 5.33(4.61,6.16) | <0.001 | 6.04(5.18,7.05) | <0.001 | 5.78(4.80,6.96) | <0.001 |
| WHO standard^b^ | General obesity status change^c^ | NN | 1(Ref.) |  | 1(Ref.) |  | 1(Ref.) |  | 1(Ref.) |  |
|  |  | YN | 2.71(2.09,3.51) | <0.001 | 2.56(1.98,3.33) | <0.001 | 2.52(1.94,3.29) | <0.001 | 2.67(1.94,3.67) | <0.001 |
|  |  | NY | 4.10(2.85,5.88) | <0.001 | 3.86(2.69,5.56) | <0.001 | 3.95(2.71,5.74) | <0.001 | 3.61(2.16,6.04) | <0.001 |
|  |  | NN | 4.58(4.05,5.18) | <0.001 | 4.43(3.91,5.03) | <0.001 | 4.71(4.13,5.38) | <0.001 | 4.90(4.17,5.76) | <0.001 |
| a ITOF standard: the International Obesity Task Force (IOTF) standard.  b WHO standard: the World Health Organization (WHO) standard.  c NN: non-obese at baseline and non-obese at follow-up; NY: non-obese at baseline and obese at follow-up; YN: obese at baseline and non-obese at follow-up; YY: obese at baseline and obese at follow-up.  Model 1 is the crude model. Model 2 is adjusted for age and gender. Model 3 is further adjusted for province, and area. Model 4 is further adjusted for fruits consumption, vegetable consumption, sugar-sweetened beverage intake, vigorous and moderate physical activity. | | | | | | | | | | |
