# Supplementary material for: Impact of short-term change of adiposity on risk of high blood pressure in children: Results from a follow-up study in China
Source: PLoS One. 2021 Sep 10;16(9):e0257144. doi: 10.1371/journal.pone.0257144 (PMC8432865; doi:10.1371/journal.pone.0257144)
Supplement: S7 Table — (DOCX) [file pone.0257144.s007.docx]

| **S7 Table. Association between risk of high blood pressure and different quintile of BMI change in the obese children based on an international definition of child obesity*** | | | | | | |
| --- | --- | --- | --- | --- | --- | --- |
| Obesity definition | Variables | Group | Model 1 | | Model 2 | |
|  |  |  | OR(95%CI) | *P* | OR(95%CI) | *P* |
| ITOF standard^a^ | BMI change (kg/m^2^) | Quintile 1(≤-0.84) | 0.52(0.36,0.76) | 0.001 | 0.47(0.32,0.69) | <0.001 |
|  |  | Quintile 2(-0.84~-0.12) | 0.59(0.41,0.86) | 0.005 | 0.55(0.37,0.80) | 0.002 |
|  |  | Quintile 3(-0.12~0.41) | 0.66(0.46,0.94) | 0.022 | 0.66(0.46,0.96) | 0.030 |
|  |  | Quintile 4(0.41~1.01) | 0.84(0.59,1.19) | 0.319 | 0.83(0.58,1.18) | 0.298 |
|  |  | Quintile 5(>1.01) | 1(Ref.) |  | 1(Ref.) |  |
| WHO standard^b^ | BMI change (kg/m^2^) | Quintile 1(≤-0.888) | 0.64(0.47,0.89) | 0.035 | 0.64(0.47,0.89) | 0.007 |
|  |  | Quintile 2(-0.888~-0.207) | 0.65(0.47,0.9) | 0.021 | 0.65(0.47,0.9) | 0.009 |
|  |  | Quintile 3(-0.207~0.315) | 0.79(0.58,1.08) | 0.102 | 0.79(0.58,1.08) | 0.137 |
|  |  | Quintile 4(0.315~0.935) | 1.01(0.75,1.37) | 0.999 | 1.01(0.75,1.37) | 0.934 |
|  |  | Quintile 5(>0.935) | 1(Ref.) |  | 1(Ref.) |  |
| a ITOF standard: the International Obesity Task Force (IOTF) standard.  b WHO standard: the World Health Organization (WHO) standard.  Model 1 is the crude model. Model 2 is adjusted for age, gender, province, and area. BMI: body mass index. WHtR: waist-to-height ratio. | | | | | | |
