# Supplementary material for: Impact of short-term change of adiposity on risk of high blood pressure in children: Results from a follow-up study in China
Source: PLoS One. 2021 Sep 10;16(9):e0257144. doi: 10.1371/journal.pone.0257144 (PMC8432865; doi:10.1371/journal.pone.0257144)
Supplement: S8 Table — (DOCX) [file pone.0257144.s008.docx]

| **S8 Table. Association between risk of high blood pressure and different quintile of BMI SDS change in the obese children** | | | | | |
| --- | --- | --- | --- | --- | --- |
| Variables | Group | Model 1 | | Model 2 | |
|  |  | OR (95%CI) | *P* | OR(95%CI) | *P* |
| BMI SDS change | Quintile 1(≤-0.38) | 0.91(0.79,1.05) | 0.197 | 0.68(0.59,0.79) | <0.001 |
|  | Quintile 2(-0.38~-0.21) | 1.01(0.87,1.18) | 0.906 | 0.87(0.74,1.01) | 0.072 |
|  | Quintile 3(-0.21~-0.08) | 1.04(0.9,1.22) | 0.574 | 0.98(0.84,1.14) | 0.782 |
|  | Quintile 4(-0.08~0.05) | 1.03(0.88,1.19) | 0.752 | 0.98(0.84,1.15) | 0.821 |
|  | Quintile 5(>0.05) | 1(Ref.) |  | 1(Ref.) |  |
| Model 1 is the crude model. Model 2 is adjusted for age, gender, province, and area. BMI: body mass index. SDS: standard deviation score. | | | | | |
