# Supplementary figures and images for: Impact of short-term change of adiposity on risk of high blood pressure in children: Results from a follow-up study in China
Source: PLoS One. 2021 Sep 10;16(9):e0257144. doi: 10.1371/journal.pone.0257144 (PMC8432865; doi:10.1371/journal.pone.0257144)

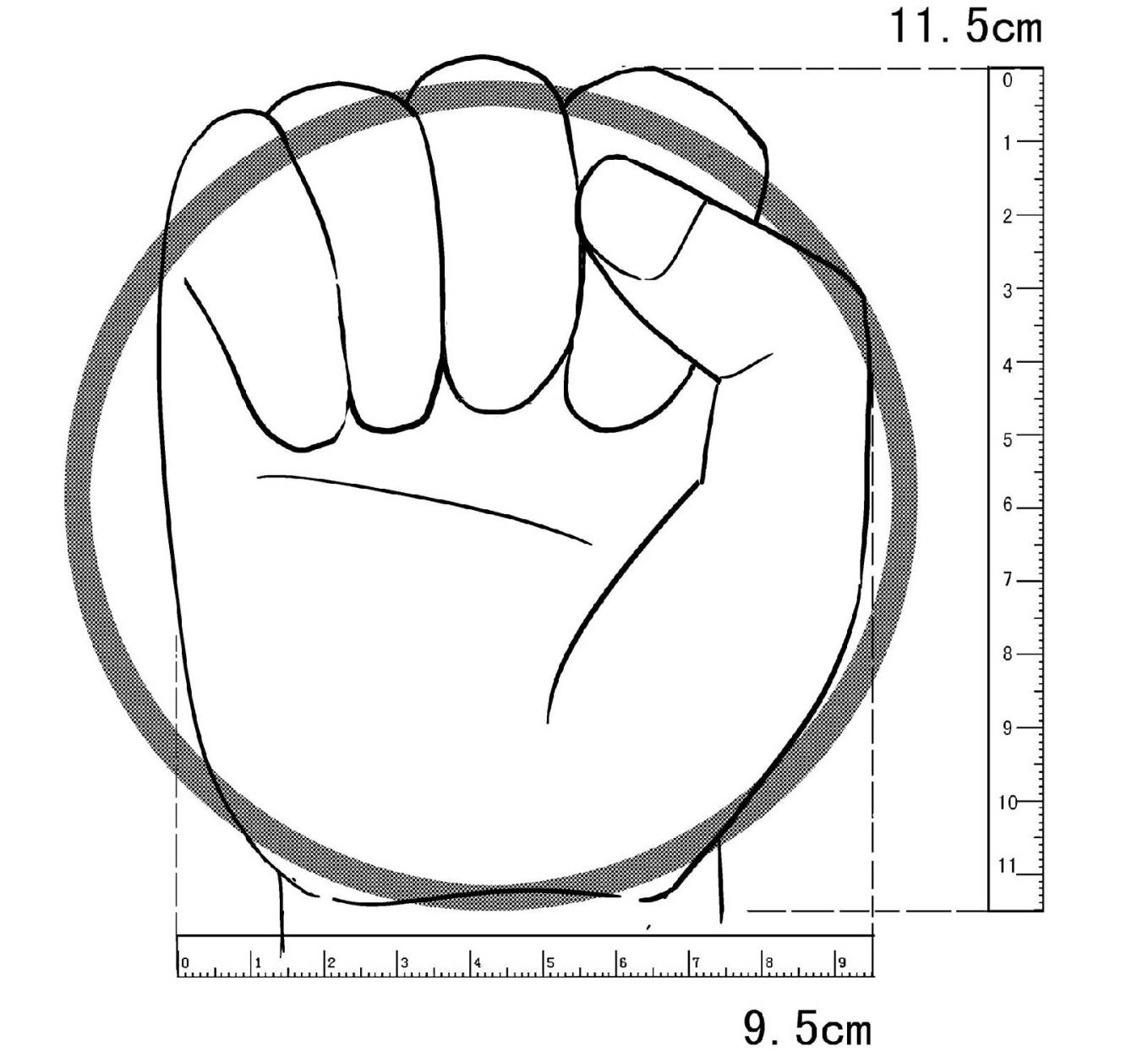


**S1 Fig.** The size of one serving of fruit or vegetable (as the size of a fist of ordinary adult).

Supplement: S1 Fig — (DOCX) [file pone.0257144.s009.docx]
