# Supplementary material for: Impact of short-term change of adiposity on risk of high blood pressure in children: Results from a follow-up study in China
Source: PLoS One. 2021 Sep 10;16(9):e0257144. doi: 10.1371/journal.pone.0257144 (PMC8432865; doi:10.1371/journal.pone.0257144)
Supplement: S2 Fig — (DOCX) [file pone.0257144.s010.docx]

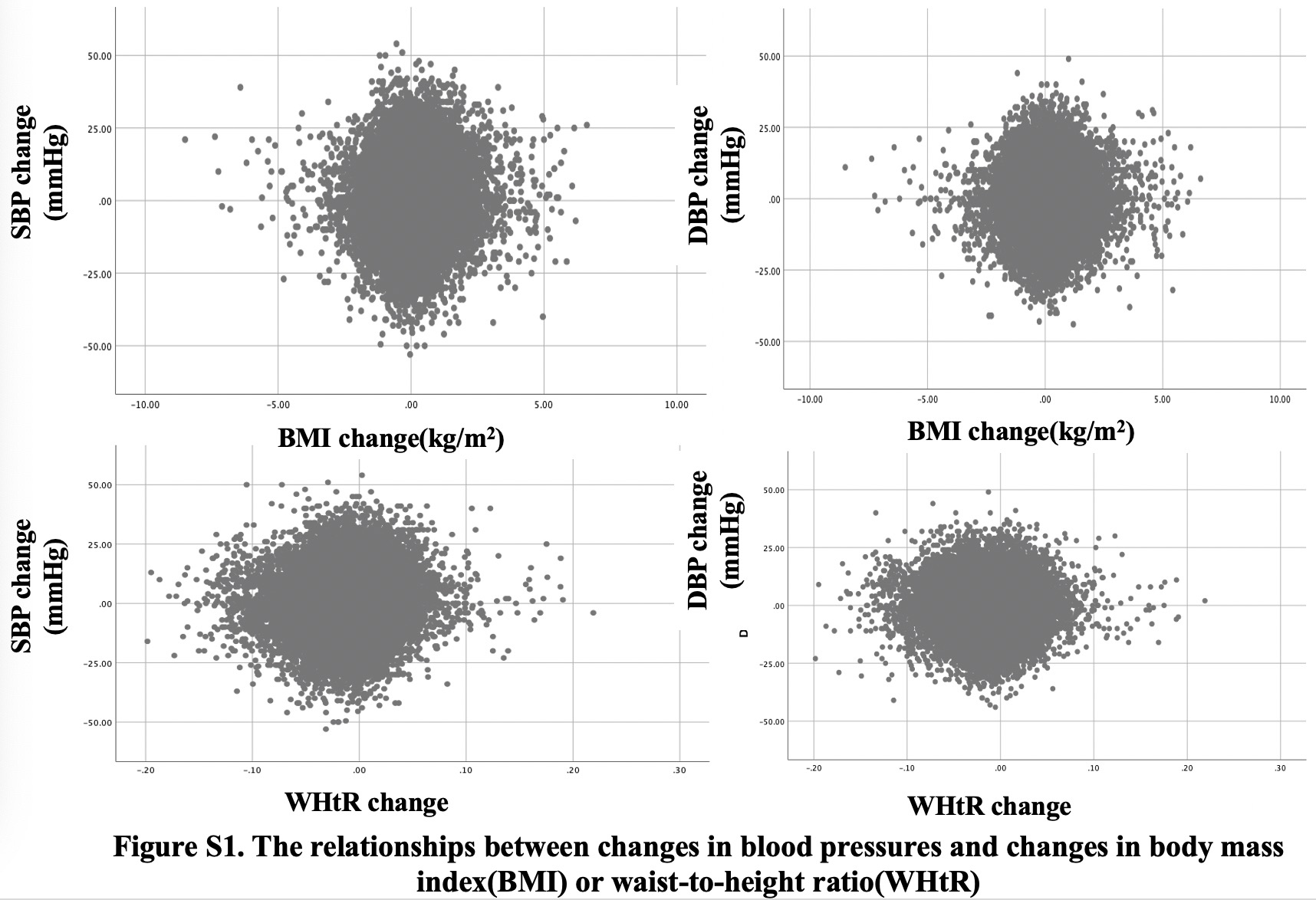


**S2 Fig.** The relationships between changes in blood pressure and changes in body mass index (BMI) or waist-to-height ratio (WHtR)
