# Supplementary material for: Impact of short-term change of adiposity on risk of high blood pressure in children: Results from a follow-up study in China
Source: PLoS One. 2021 Sep 10;16(9):e0257144. doi: 10.1371/journal.pone.0257144 (PMC8432865; doi:10.1371/journal.pone.0257144)
Supplement: S1 File — (DOC) [file pone.0257144.s011.doc]

**Impact of short-term change of adiposity on risk of high blood pressure in children: Results from a follow-up study in China.**

Yi-de Yang^1,2*#^, Ming Xie^1#^, Yuan Zeng^1#^, Shuqian Yuan^1^, Yanhui Dong^2^, Zhiyong Zou^2^, Bin Dong^2*^, Zhenghe Wang^3^, Xiangli Ye^1^, Xiuqin Hong^1^, Qiu Xiao^4^, Jun Ma^2^

^1^ Key Laboratory of Molecular Epidemiology of Hunan Province, School of Medicine, Hunan Normal University, Changsha, 410006, China

^2^ Institute of Child and Adolescent Health, School of Public Health, Peking University Health Science Center, Beijing, 100191, China;

^3^ Department of Epidemiology, School of Public Health, Southern Medical University, Guangzhou, Guangdong, 510515, China

^4^ College of Information Science and Engineering, Hunan Normal University, Changsha, 410081, China

**Running title:** Short-term adiposity change and HBP

* **Corresponding authors:** **Yi-de Yang***,* Email: [yangyide@hunnu.edu.cn](mailto:yangyide@hunnu.edu.cn), School of Medicine, Hunan Normal University, Changsha, 410081, China and **Bin Dong**, email: [bindong@bjmu.edu.cn](mailto:bindong@bjmu.edu.cn), Institute of Child and Adolescent Health, School of Public Health, Peking University Health Science Center, Beijing, 100191, China. Phone number: +86 10 82801624, fax: +86 10 82801178.

# These authors contributed equally to this work.

**Supplementary file 2 (English version of Questionnaire)**

**(The questions in blue were used as behavioral covariates in our study)**

**Questionnaire about dietary factors and physical activities in children and adolescents**

**(For Grade 1-3 students of elementary school, the questionnaire should be finished by the parents)**

Dear participants,

We would like to investigate your dietary factors and physical activities levels in order to improve your health and develop good dietary and exercise habits. This is not an exam. There are no true answers, and we hope that you could answer these questions honestly. Your answers are strictly confidential. We hope that you could read and answer every question carefully and independently.

Please mark the option with “ ” as the following example question(Q1). Every question is a single topic selection. Please fill in the blanks “ ” with your answer as the following example question(Q2). The information about your parents and the questions you do not understand should be answered by your parents.

Example:

**Q1. Do you like reading？ [1] like [2] dislike**

**Q2. During the past week you smoked cigarette for ___2___ days.**

Name：____________________

Sex：[1] male [2] female

School name：____________________

Grade：____________________

Class：____________________

Birthdate：________________ (YYYY/MM/DD)

Investigation Date：________________(YYYY/MM/DD)

**Section 1. Basic information**

1.Who will finish the questionnaire：

[1]by yourself(student) [2]by parents [3]together with parents [4]together with grandparents [5]others

2.Are you living in the school in this semester？

[1] Yes [2] No

**Section 2. Dietary behaviors**

1. During the last 7 days, how many days did you eat fruit? days

During the days you ate fruit, how many servings of fruit have eaten per day? servings

(One serving of fruit is equivalent to the size of an adult’s fist, please see the figure on the last page of this questionnaire.)

2. During the last 7 days, how many days did you eat vegetable (not including pickles)? days

During the days you ate vegetable, how many servings of fruit have eaten per day? servings

(One serving of fruit is equivalent to the size of an adult’s fist, please see the figure on the last page of this questionnaire.)

3. During the last 7 days, how many days did you drink sugar-sweetened beverage? days

(Including Coca Cola, sprite, orange juice, Nutrition express, Red Bull et al)

During the days you drank sugar-sweetened beverage, how many cups have drunk per day? cups

(One cup is equivalent to 250ml or half bottle.)

1. How many days did you eat breakfast in the last 7 days? days
2. How many days did you eat milk (Fresh milk, yogurt or milk powder) in the last 7 days? days
3. How many days did you eat high calorie food (cakes, chocolate, candy, fried chips) in the last 7 days?

days

1. How many days did you eat fried food (such as fried chicken, fried bread stick, deep-fried cake, deep-fried pancake) in the last 7 days?

days

1. How many times did you eat in a restaurant or dining-hall in the last 7 days? Times
2. How many times did you eat western fast food (KFC, McDonalds and Pizza et al) in the last 30 days?

Times

1. When you are eating at home, the size of bowl you used compared with an adult’ bowl?

[1] smaller than the adult’s bowl [2] same size [3] bigger than the adult’s bowl

11. Comparing with the eating speed with a classmate with you sex:

[1] slower than others [2] same speed [3] faster than others

**Section 3. Physical activities related behaviors**

1. During the last 7 days, how many days did spend to do vigorous physical activities? (*The* vigorous *physical activities made you sweat and out of breath, including playing basketball, football, running, swimming, fast bicycling, dancing or other similar activities.*)

days On these days, how long did you spent on the vigorous physical activities per day？

Each day ______ hour______ minutes

1. During the last 7 days, how many days did spend to do moderate physical activities per day? (*The physical activities did not make you sweat or out of breath, including slow bicycling, play ping-pong, play badminton, skating and cleaning the floor. Not including walking.*)

days On these days, how long did you spent on the moderate physical activities per day？

Each day ______ hour______ minutes

1. During the last 7 days, how many days did you walk? (*Including walking at home or school, commuting between school and home, and walking for exercise.*)

days On these days, how long did you spent on walking per day？

Each day ______ hour______ minutes

1. During the last 7 days, how long did spend on sitting or lying down (not including sleeping time) per day?

Each day______ hour______ minutes

Among this: How long for after school homework？ Each day______ hour______ minutes

How long for watching TV？ Each day______ hour______ minutes

How long for play computer or video game？ Each day______ hour______ minutes

1. How long did you regularly spend on outdoor activities per day in the last 7 days?

[1] <1 hour [2]1～2hours(not including 2hours) [3] 2～4hours [4]＞4hours [5]don’t know

1. How long did you regularly get sunlight per day when it was sunny in the last 7 days?

[1]＜30min [2]31～59min [3]1～2hours(not including 2hours) [4]2～4hours [5]＞4hours [6] don’t know


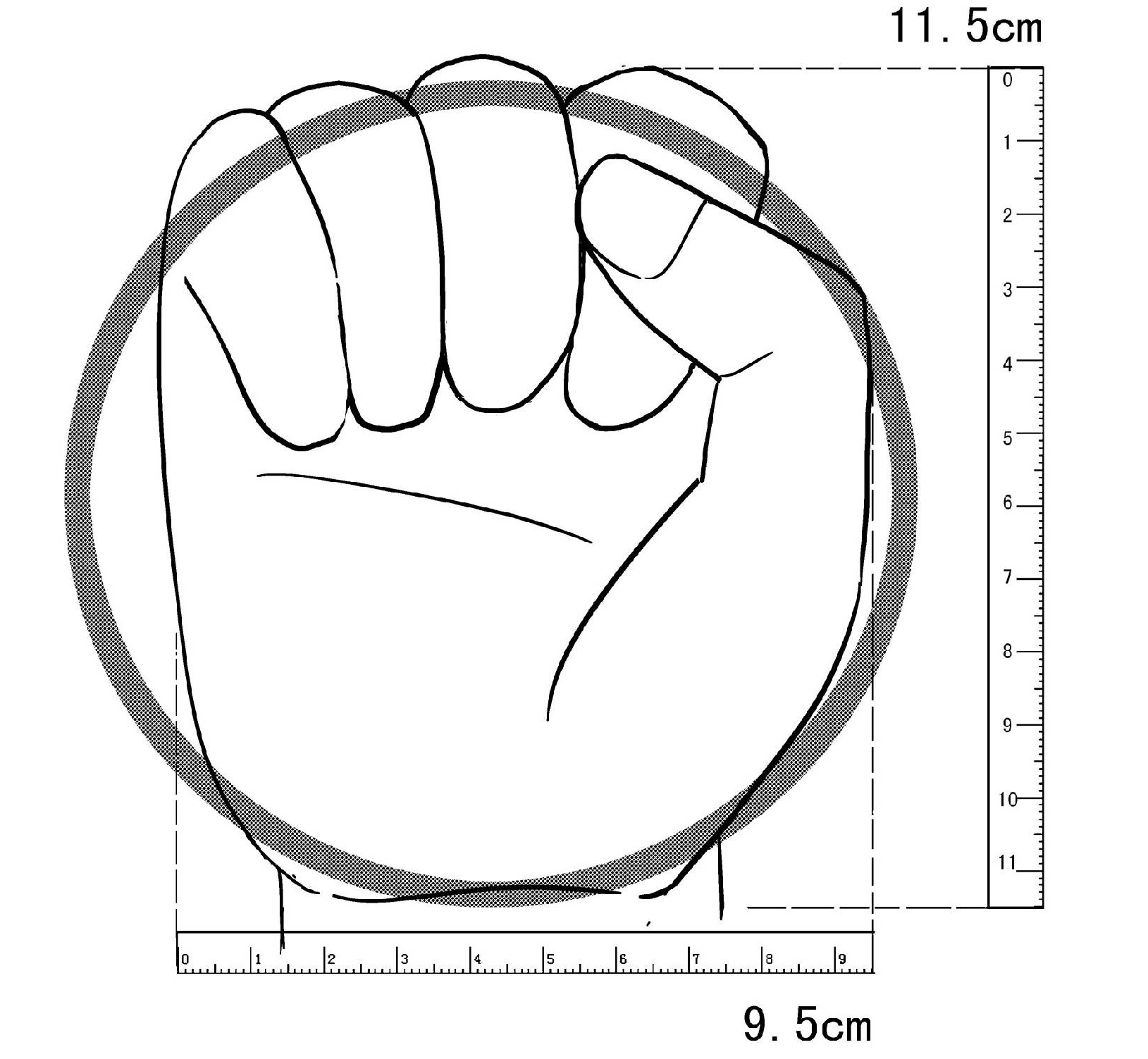


**Figure S1.** The size of one serving of fruit or vegetable (as the size of a fist of ordinary adult).
