# Supplementary material for: Impact of short-term change of adiposity on risk of high blood pressure in children: Results from a follow-up study in China
Source: PLoS One. 2021 Sep 10;16(9):e0257144. doi: 10.1371/journal.pone.0257144 (PMC8432865; doi:10.1371/journal.pone.0257144)
Supplement: Attachment — Submitted filename: PONE-D-21-04822 - Impact of short-term change of adiposity on risk of high blood pressure in children, follow-up study in China.pdf [file pone.0257144.s012.pdf]

PLOS ONE

Impact of short-term change of adiposity on risk of high blood pressure in children: Results from a follow-up study in China.

--Manuscript Draft--

**Manuscript Number:**	PONE-D-21-04822
**Article Type:**	Research Article
**Full Title:**	Impact of short-term change of adiposity on risk of high blood pressure in children: Results from a follow-up study in China.
**Short Title:**	Short-term adiposity change and HBP
**Corresponding Author:**	Yi-de Yang<br>Institute of Child and Adolescent,School of Public Health<br>Beijing, CHINA
**Keywords:**	blood pressure; Children; general obesity; abdominal obesity; change
**Abstract:**	This study aimed to examine the impact of short-term adiposity change on risk of high blood pressure (HBP), and to assess the low limit range of body mass index (BMI) and waist-to-height ratio (WHtR) reduction proposed to decrease the HBP risk in children. Children were longitudinally surveyed at baseline and after a short-term follow-up. General obesity (GOB) is categorized by age and gender-specific BMI cut-off points, abdominal obesity (AOB) by WHtR. Logistic regression model was used to estimate relations between adiposity change and HBP risk with adjustment of covariates. A total of 28,288 children (mean baseline age of 11.22±3.31 years) were involved with follow-up of 6.88±1.20 months. After the follow-up, 9.4% of the children had persistent general obesity (GOB), 2.8% converted from GOB to non-GOB, 0.9% had newly developed GOB. When compared with children remained non-GOB, children with continuous GOB status, newly developed GOB, converting from GOB to non-GOB had 5.03-fold (95%CI: 4.32~5.86), 3.35-fold (95%CI: 1.99~5.65), 2.72-fold (2.03~3.63) HBP risk, respectively. Similar findings were observed for abdominal obesity(AOB). Reduction of 0.21-0.88 kg/m 2 of baseline BMI(0.86-3.59%) or 0.009-0.024 of baseline WHtR(1.66-4.42%) in GOB or AOB children, respectively, was associated with significant decrease in HBP risk. Children with persistent obesity, newly developed obesity, or converting from obese to non-obese had significantly higher HBP risk. For children with GOB or AOB, reduction of <3.6% in BMI or <4.5% in WHtR could decrease the HBP risk.
**Order of Authors:**	Yi-de Yang
	Ming Xie
	Shuqian Yuan
	Yuan Zeng
	Yanhui Dong
	Zhiyong Zou
	Bin Dong
	Zhenghe Wang
	Xiangli Ye
	Xiuqin Hong
	Qiu Xiao
	Jun Ma
**Additional Information:**	
**Question**	**Response**
**Financial Disclosure**	The funders had no role in study design, data collection and analysis, decision to

Powered by Editorial Manager® and ProduXion Manager® from Aries Systems Corporation

Enter a financial disclosure statement that describes the sources of funding for the work included in this submission. Review the submission guidelines for detailed requirements. View published research articles from *PLOS ONE* for specific examples.<br><br>This statement is required for submission and **will appear in the published article** if the submission is accepted. Please make sure it is accurate.<br><br>**Unfunded studies**<br>Enter: *The author(s) received no specific funding for this work.*<br><br>**Funded studies**<br>Enter a statement with the following details:<br>• Initials of the authors who received each award<br>• Grant numbers awarded to each author<br>• The full name of each funder<br>• URL of each funder website<br>• Did the sponsors or funders play any role in the study design, data collection and analysis, decision to publish, or preparation of the manuscript?<br>• **NO** - Include this sentence at the end of your statement: *The funders had no role in study design, data collection and analysis, decision to publish, or preparation of the manuscript.*<br>• **YES** - Specify the role(s) played.<br><br>* typeset	publish, or preparation of the manuscript.
**Competing Interests**<br><br>Use the instructions below to enter a competing interest statement for this submission. On behalf of all authors, disclose any competing interests that could be perceived to bias this work—acknowledging all financial support and any other relevant financial or non-financial competing interests.<br><br>This statement is required for submission and **will appear in the published article** if	The authors have declared that no competing interests exist.

Powered by Editorial Manager® and ProduXion Manager® from Aries Systems Corporation

the submission is accepted. Please make sure it is accurate and that any funding sources listed in your Funding Information later in the submission form are also declared in your Financial Disclosure statement.<br><br>View published research articles from *PLOS ONE* for specific examples.<br><br>**NO authors have competing interests**<br><br>Enter: *The authors have declared that no competing interests exist.*<br><br>**Authors with competing interests**<br><br>Enter competing interest details beginning with this statement:<br><br>*I have read the journal's policy and the authors of this manuscript have the following competing interests: [insert competing interests here]*<br><br>* typeset	
**Ethics Statement**<br><br>Enter an ethics statement for this submission. This statement is required if the study involved:<br><br>• Human participants<br>• Human specimens or tissue<br>• Vertebrate animals or cephalopods<br>• Vertebrate embryos or tissues<br>• Field research<br><br>Write "N/A" if the submission does not require an ethics statement.<br><br>General guidance is provided below. Consult the submission guidelines for detailed instructions. **Make sure that all information entered here is included in the Methods section of the manuscript.**	All procedures performed in the present study were in accordance with the Declaration of Helsinki and were approved by the Medical Ethical Committee of the Peking University Health Science Center (NO.IRB0000105213034). The author obtained permission to use the dataset from Institute of Child and Adolescent health, Peking University and the secondary analysis of the dataset without identifiable information was approved by the Ethical Committee of Hunan Normal University(NO.2019-88) and by Southern Medical University(NO.IRB00001052-20014). Before investigation, written informed consent to participate in the study was obtained from both the children and their parents or legal guardians. The written informed consent was obtained for all forms of personally identifiable data including biomedical, clinical, and biometric data. Also, all participants had the right to withdraw from the study at any time and for any reason. All participants, together with their parents or legal guardians, had been informed that this investigation would be performed anonymously and confidentially, and their privacy would be protected and respected.

Powered by Editorial Manager® and ProduXion Manager® from Aries Systems Corporation

**Format for specific study types**<br><br>**Human Subject Research (involving human participants and/or tissue)**<br>• Give the name of the institutional review board or ethics committee that approved the study<br>• Include the approval number and/or a statement indicating approval of this research<br>• Indicate the form of consent obtained (written/oral) or the reason that consent was not obtained (e.g. the data were analyzed anonymously)<br><br>**Animal Research (involving vertebrate animals, embryos or tissues)**<br>• Provide the name of the Institutional Animal Care and Use Committee (IACUC) or other relevant ethics board that reviewed the study protocol, and indicate whether they approved this research or granted a formal waiver of ethical approval<br>• Include an approval number if one was obtained<br>• If the study involved *non-human primates*, add *additional details* about animal welfare and steps taken to ameliorate suffering<br>• If anesthesia, euthanasia, or any kind of animal sacrifice is part of the study, include briefly which substances and/or methods were applied<br><br>**Field Research**<br><br>Include the following details if this study involves the collection of plant, animal, or other materials from a natural setting:<br>• Field permit number<br>• Name of the institution or relevant body that granted permission	
**Data Availability**<br><br>Authors are required to make all data underlying the findings described fully available, without restriction, and from the time of publication. PLOS allows rare exceptions to address legal and ethical concerns. See the PLOS Data Policy and FAQ for detailed information.	No - some restrictions will apply

A Data Availability Statement describing where the data can be found is required at submission. Your answers to this question constitute the Data Availability Statement and **will be published in the article**, if accepted.<br><br>**Important:** Stating 'data available on request from the author' is not sufficient. If your data are only available upon request, select 'No' for the first question and explain your exceptional situation in the text box.<br><br>Do the authors confirm that all data underlying the findings described in their manuscript are fully available without restriction?	
**Describe where the data may be found in full sentences. If you are copying our sample text, replace any instances of XXX with the appropriate details.**<br><br>• If the data are **held or will be held in a public repository**, include URLs, accession numbers or DOIs. If this information will only be available after acceptance, indicate this by ticking the box below. For example: *All XXX files are available from the XXX database (accession number(s) XXX, XXX.).*<br>• If the data are all contained **within the manuscript and/or Supporting Information files**, enter the following: *All relevant data are within the manuscript and its Supporting Information files.*<br>• If neither of these applies but you are able to provide **details of access elsewhere**, with or without limitations, please do so. For example:<br><br>*Data cannot be shared publicly because of [XXX]. Data are available from the XXX Institutional Data Access / Ethics Committee (contact via XXX) for researchers who meet the criteria for access to confidential data.*<br><br>*The data underlying the results presented in the study are available from (include the name of the third party*	The data analyzed in the present study are not publicly available due to data management rules of Peking University and Hunan Normal University, China, but are available from the corresponding author on reasonable request.

and contact information or URL).<br>• This text is appropriate if the data are owned by a third party and authors do not have permission to share the data.<br><br>* typeset	
Additional data availability information:	

Manuscript
Click here to access/download;Manuscript;manuscript 20210212.docx

Impact of short-term change of adiposity on risk of high blood pressure in children: Results from a follow-up study in China.

Yide Yang[1,2], Ming Xie[1], Shuqian Yuan[1], Yuan Zeng[1], Yanhui Dong[2], Zhiyong Zou[2], Bin Dong[2*], Zhenghe Wang[3], Xiangli Ye[1], Xiuqin Hong[1], Qiu Xiao[4*], Jun Ma[2]

[1] Key Laboratory of Molecular Epidemiology of Hunan Province, School of Medicine, Hunan Normal University, Changsha, 410006, China

[2] Institute of Child and Adolescent Health, School of Public Health, Peking University Health Science Center, Beijing, 100191, China;

[3] Department of Epidemiology, School of Public Health, Southern Medical University, Guangzhou, Guangdong, 510515, China

[4] College of Information Science and Engineering, Hunan Normal University, Changsha, 410081, China

* **Corresponding authors: Qiu Xiao**, Email: xiaoqiu@hunnu.edu.cn, College of Information Science and Engineering, Hunan Normal University, Changsha, 410081, China and **Bin Dong**, email: bindong@bjmu.edu.cn, Institute of Child and Adolescent Health, School of Public Health, Peking University Health Science Center, Beijing, 100191, China. Phone number: +86 10 82801624, fax: +86 10 82801178.

Running title: Short-term adiposity change and HBP

Abbreviations

BMI: Body mass index; GOB, general obesity; AOB, abdominal obesity; WHtR, waist-to-height ratio; BP, blood pressure; SBP, systolic blood pressure; DBP, diastolic blood pressure; HBP, high blood pressure; SSB: sugar-sweetened beverage.

Financial disclosure

The authors have no conflicts of interest to disclose.

Potential conflict of interest

The authors have declared that no competing interests exist.

Abstract

This study aimed to examine the impact of short-term adiposity change on risk of high blood pressure (HBP), and to assess the low limit range of body mass index (BMI) and waist-to-height ratio (WHtR) reduction proposed to decrease the HBP risk in children. Children were longitudinally surveyed at baseline and after a short-term follow-up. General obesity (GOB) is categorized by age and gender-specific BMI cut-off points, abdominal obesity (AOB) by WHtR. Logistic regression model was used to estimate relations between adiposity change and HBP risk with adjustment of covariates. A total of 28,288 children (mean baseline age of 11.22±3.31 years) were involved with follow-up of 6.88±1.20 months. After the follow-up, 9.4% of the children had persistent general obesity (GOB), 2.8% converted from GOB to non-GOB, 0.9% had newly developed GOB. When compared with children remained non-GOB, children with continuous GOB status, newly developed GOB, converting from GOB to non-GOB had 5.03-fold (95%CI: 4.32~5.86), 3.35-fold (95%CI: 1.99~5.65), 2.72-fold (2.03~3.63) HBP risk, respectively. Similar findings were observed for abdominal obesity(AOB). Reduction of 0.21-0.88 kg/m^2 of baseline BMI(0.86-3.59%) or 0.009-0.024 of baseline WHtR(1.66-4.42%) in GOB or AOB children, respectively, was associated with significant decrease in HBP risk. Children with persistent obesity, newly developed obesity, or converting from obese to non-obese had significantly higher HBP risk. For children with GOB or AOB, reduction of <3.6% in BMI or <4.5% in WHtR could decrease the HBP risk.

Key words: blood pressure; children; general obesity; abdominal obesity; change

Introduction

The prevalence and magnitude of pediatric high blood pressure(HBP) increased dramatically in recent years, which pose a heavy burden for cardiovascular diseases (CVDs) prevention[1]. As the most important cause of CVDs, HBP composed the largest number of attributable disability-adjusted life-years (DALYs) in China[2].

The sharp increase of overweight and obesity prevalence contributes greatly to the epidemic of HBP among children[3]. The association between obesity and blood pressure (BP) levels or hypertension has been demonstrated in numerous cross-sectional studies[4-6]. Also, intervention studies against obesity showed that weight management will additionally benefit ~~for~~ BP profile[7]. But in a natural life course, it remains unclear whether the change of general or abdominal fat accumulation status without intervention would impact the risk of HBP in childhood.

In addition, a national secular trend study of BP and obesity among Chinese children showed that the magnitude of impact of obesity on HBP increase sharply in recent years[1]. Therefore, in a natural life course the benefit of conversion of obesity to non-obesity and the risk of newly developed obesity or persistent obesity during childhood may also vary over time. Accordingly, we made a hypothesis that the conversion of obesity to non-obesity could be beneficial for reducing the risk of HBP, and the newly developed or persistent obesity is detrimental to the BP profile among childhood.

Previous weight management study among overweight children show that ~~only 5% reduction~~ reduction of just 5% of body mass index (BMI) is associated with improvements in lipid profiles and insulin

sensitivity in six months [8], and a meta-analysis of lifestyle intervention studies showed that BMI standard deviation score (SDS) reduction of >1 SDS is associated with better BP profiles[7]. However, in a natural life course without intervention, for obese children the minimum range of adiposity reduction proposed to reduce the HBP risk is not known now. Hence, our study aimed to identify how the general obesity (GOB) or abdominal obesity (AOB) status will change in a short-term follow up, and to demonstrate the relationship between different obesity status change and BP profiles, including BP levels and risk of HBP in children, and also assess the low limit range for reduction of adiposity parameters (such as BMI and waist-to-height ratio) in a natural growth process proposed to be beneficial for reducing HBP risk in obese children.

Subjects and Methods

Study Design and Participants

Data were deprived from a multi-center school-based obesity intervention program which involved more than 70,000 children aged 6–17 years based on a random sampling method from 7 provinces in China at autumn of 2013. A detailed description of the program was published before[9]. To study the natural change of obesity status and its associations with risk of HBP, we only included participants of the control group of this intervention program. A total of 28,288 participants without any intervention were involved in the present study, with complete data of baseline and the follow up anthropometric data (height, weight, waist circumference and blood pressure). This study was approved by the Medical Ethical Committee of the Peking University Health Science Center (IRB0000105213034). Written

informed consent was obtained from all children and their parents.

Measurements and Definitions

Anthropometric measurements, such as height, weight, waist circumference and blood pressure were measured according to standardized protocols. Height was measured to the nearest 0.1cm with shoes. Weight was measured to the nearest 0.1 kg by a Lever type weight scale. Every indicator was measured twice, and the mean value of the two measurements were used for final analyses. BMI (kg/m^2) was calculated as weight(kg) divided by height(m) squared. Participants were categorized as generally obese if the BMI was larger than the age-and gender-specific cut-off points, according to the standard “BMI Reference for Screening for overweight and obesity among school-age children and adolescents” published by National Health Commission of the People‘s Republic of China[10](**S1 Table**).Waist circumference was measured to the nearest 0.1cm, at the midway between the superior border of participant’s iliac crest and the lowest rib with a flexible nylon tape. Waist-to-height ratio (WHtR) was calculated as waist circumference (cm) divided by height (cm). Participants were categorized as abdominally obese if their WHtR was not less than 0.5[11, 12].

BP was measured by a mercury sphygmomanometer and it was measured two times, with at least five-minute interval between each measurement. If the measured difference was >10 mmHg, measurement was repeated until the final two measures differed <10 mmHg, and the mean of the final two measures was used in analyses. Children were asked to remain quiet and to sit still while each reading was being taken. Systolic blood pressure (SBP) was defined as the onset of “tapping” Korotkoff sound (K1), and diastolic blood pressure (DBP) was

defined as the fifth Korotkoff sound (K5). High BP is defined as SBP and/or DBP ≥ the age-, gender- and height-specific 95th percentile[13].

Covariates

Data of demographic information (birth date, gender and area), fresh fruit and vegetable consumption, sugar-sweetened beverage (SSB) intake, and time of vigorous or moderate physical activities at baseline were collected by a standard questionnaire[14, 15](**S1 File**). Area was categorized as rural area and urban area of the school location. ~~The~~ frequency (days per week) and amount (servings) of fruits and vegetables, separately, consumed in the past week were investigated. The average of daily intake of vegetables was calculated as follows: average daily fresh vegetable consumption = [frequency × (servings of vegetable consumed in each of these days)]/7[15],and the size of one serving of fruit or vegetable is as the size of a fist of ordinary adult (**S1 Fig.**). For the average daily fresh fruit consumption, similar equation was used as well. For SSB intake, every child answered two questions of the frequency of the intake and usual portion size(cups) of all SSB, including orange juice, Coca-Cola, Nutrition Express, Sprite and Red Bull (one cup=250 ml). We combined these items to calculate the weekly SSB intake[16]. Physical activities included vigorous physical activities (activities which obviously increase the breathing and heart rate, for example, running, football, basketball et al.), and moderate physical activities (activities which increase the breathing and heart rate to some extent, for example bicycle, table tennis, calisthenics and badminton et al.). ~~The~~ frequency (days per week) and time (specific hours and minutes for each of these days) spent on these activities in the past week were investigated. We used equation: average daily

time = [days × (time in each of those days)]/7, to calculate specific average time each day the children participated in vigorous physical activities or moderate physical activities[1].

Statistical analysis

Means with standard deviations were used to describe numerical variables. Frequencies were used to describe the categorical variables. Chi-square tests were used to compare the categorical variables between different groups, independent t tests were conducted quantitative variables between two groups, one-way ANOVA were used to compare quantitative variables in multiple groups. Adjusted means and 95% confidence interval of BP levels and BP changes were estimated by multivariate general linear model with adjusting gender, age, BMI, area, fruit consumption, vegetable consumption, SSB intake, and physical activity. Multivariable logistic regression model was used to examine the association between different obesity status change and the risk of HBP during six-month follow up adjusted for potential baseline covariates.

For children with GOB or AOB at baseline, we categorized their adiposity parameter (BMI or WHtR) change during the follow-up as quintiles (Quintile 1: $\leq P_{20}$, Quintile 2: P_{20}~P_{40}, Quintile 3: P_{40}~P_{60}, Quintile 4: P_{60}~P_{80}, Quintile 5: $>P_{80}$), and compared the risk of HBP in different quintiles with multivariate logistic models.

We additionally conducted a series of sensitivity analyses to test the robustness of the results: (1) We added height change from baseline to follow up survey in the models to examine the growth effect on the associations between obesity status change and risk of HBP. (2) We included the baseline HBP status as a covariate in the models to investigate the influence on

the associations. (3) For children with GOB or AOB at baseline, we also categorized their adiposity parameter (BMI or WHtR) change during the follow-up as ten equal groups (Group 1: $\leq P_{10}$, Group 2: P_{10}~P_{20}, Group 3: P_{20}~P_{30}, Group 4: P_{30}~P_{40}, Group 5: P_{40}~P_{50}, Group 6: P_{50}~P_{60}, Group 7: P_{60}~P_{70}, Group 8: P_{70}~P_{80}, Group 9: P_{80}~P_{90} and Group 10: $>P_{90}$) and compared the risk of HBP in different groups with multivariate logistic models.

All *P* values were two-sided and *P* value of <0.05 were considered as statistically significant. SPSS for Windows (version 20.0, SPSS Inc, Chicago, IL, USA) was used for statistical analysis.

Results

Characteristic of the study population

A total of 28,288 children aged 6-17 years were included for the present study, 51.4% were boys, with mean age of 11.22±3.31 years, and the mean follow up period was 6.88±1.20 months. At the baseline, 12.2% of the children were categorized as GOB (15.3% and 9.0% for boys and girls, respectively), 15.5% had AOB (19.0% and 11.8% for boys and girls, respectively), and 9.7% had HBP (10.5% and 8.7% for boys and girls, respectively) (**Table 1**).

At the follow-up survey, 10.3%, 12.4% and 5.8% of the participants had GOB, AOB and HBP, respectively. During the short-term follow-up period, for GOB, 86.9% of the children remained a non-GOB status, 9.4% of the children had persistent GOB, 2.8% converted from GOB to non-GOB, and 0.9% had newly developed GOB. Among the 3454 children with

GOB at baseline, 77.2% of them had persistent GOB, and the conversion rate from GOB to non-GOB was 22.8% without intervention.

While for AOB, 82.6% of the children remained a non-AOB status, 10.5% of the children remained as AOB, 4.9% converted from AOB to non-AOB and 1.9% had newly developed AOB (**Table 1**).

Table 1. General characteristics of the study population

Variables	Group	Boys (n=14542)		Girls (n=13746)		Total (N=28288)		*P* value
		N/Mean	%/SD	N/Mean	%/SD	N/Mean	%/SD	
Characteristics at the Baseline survey								
Area	urban	9520	65.50%	9035	65.70%	18555	65.60%	0.642
	rural	5022	34.50%	4711	34.30%	9733	34.40%	
Age(years)		11.13	3.26	11.32	3.35	11.22	3.31	<0.001
Height(cm)		146.19	18.33	143.96	15.4	145.11	17.01	<0.001
Weight(kg)		41.86	16.7	39.05	13.88	40.49	15.46	<0.001
BMI(kg/m^2) [a]		18.81	3.87	18.2	3.55	18.52	3.73	<0.001
Waist circumference(cm)		65.58	11.32	63.46	9.86	64.55	10.69	<0.001
WHtR[b]		0.45	0.06	0.44	0.05	0.45	0.05	<0.001
SBP(mmHg)		105.64	12.21	102.76	11.47	104.24	11.94	<0.001
DBP(mmHg)		66.82	8.84	65.65	8.34	66.25	8.62	<0.001
Fruits(servings/day)[c]		1.25	1.09	1.27	1	1.26	1.05	<0.001
Vegetables(servings/day) [c]		1.8	1.43	1.75	1.33	1.77	1.38	<0.001
SSB [d] (cup/day)[e]		0.47	0.83	0.31	0.59	0.39	0.73	<0.001
Vigorous physical activity(hour/day)		0.52	0.74	0.38	0.6	0.45	0.68	<0.001
Moderate physical activity(hour/day)		0.52	0.79	0.43	0.64	0.48	0.73	<0.001
General obesity	no	12319	84.70%	12515	91.00%	24834	87.80%	<0.001
	yes	2223	15.30%	1231	9.00%	3454	12.20%	
Abdominal obesity	no	11723	81.00%	12071	88.20%	23794	84.50%	<0.001
	yes	2754	19.00%	1612	11.80%	4366	15.50%	
HBP	no	13012	89.50%	12546	91.30%	25558	90.30%	<0.001

	yes	1530	10.50%	1200	8.70%	2730	9.70%	
Characteristics at the follow-up survey								
Age(years)		11.74	3.27	11.94	3.36	11.84	3.32	<0.001
Height(cm)		149.19	17.8	146.35	14.39	147.81	16.29	<0.001
Weight(kg)		43.66	16.99	40.66	13.92	42.2	15.65	<0.001
BMI(kg/m^2)		18.87	3.94	18.4	3.67	18.64	3.82	<0.001
Waist circumference(cm)		65.4	11.51	62.82	9.89	64.14	10.83	<0.001
WHtR		0.44	0.06	0.43	0.05	0.43	0.05	<0.001
SBP(mmHg)[f]		105.78	11.86	103.19	10.64	104.52	11.36	<0.001
DBP(mmHg)[g]		66.26	8.55	65.15	7.95	65.72	8.28	<0.001
General obesity	no	12714	87.40%	12668	92.20%	25382	89.70%	<0.001
	yes	1828	12.60%	1078	7.80%	2906	10.30%	
Abdominal obesity	no	12141	84.00%	12523	91.40%	24664	87.60%	<0.001
	yes	2320	16.00%	1184	8.60%	3504	12.40%	
HBP[h]	no	13617	93.60%	13019	94.70%	26636	94.20%	<0.001
	yes	925	6.40%	727	5.30%	1652	5.80%	
Change of obesity status from baseline to follow-up								
General obesity status change[i]	NN	12185	83.80%	12408	90.30%	24593	86.90%	0.824
	YN	529	3.60%	260	1.90%	789	2.80%	
	NY	134	0.90%	107	0.80%	241	0.90%	
	YY	1694	11.60%	971	7.10%	2665	9.40%	
Abdominal obesity status change[i]	NN	11391	79.10%	11798	86.40%	23189	82.60%	<0.001
	YN	707	4.90%	674	4.90%	1381	4.90%	
	NY	289	2.00%	248	1.80%	537	1.90%	
	YY	2021	14.00%	931	6.80%	2952	10.50%	

a BMI: body mass index. b WHtR: waist-to-height ratio. c A serving of fruit or vegetable is equivalent to 100 g. d SBBs means sugar–sweetened beverage. e A cup is equivalent to 250 ml. f SBP: systolic blood pressure. g DBP: diastolic blood pressure. h

HBP: high blood pressure. i NN: non-obese at baseline and non-obese at follow-up; NY: non-obese at baseline and obese at follow-up; YN: obese at baseline and non-obese at follow-up; YY: obese at baseline and obese at follow-up.

The BP levels and the risk of HBP in children with different obesity status change

Among children of different GOB status change groups, the baseline SBP and DBP were significantly different (*P*<0.001). ~~The~~ SBP and DBP levels at follow-up, as well as the change of SBP/DBP during the short-term follow-up, of children in different groups were also significantly different (*P*<0.05). The risk of HBP was significantly different whenever baseline or follow-up in different GOB status change groups (*P*<0.001). ~~The~~ children with continuous GOB (YY group) had the highest prevalence of HBP (17.6%) at the follow-up survey, 12.9% for newly developed GOB group (NY group), 11.2% for children who had GOB before but not now (YN group), and 4.3% for the stable non-obese children (NN group). A similar pattern was also observed for AOB (**Table 2**).

Table 2. Blood pressure level and risk of high blood pressure of different pattern of general or abdominal obesity

**Variables**		**NN**		**YN**		**NY**		**YY**		***P* value**
		**N/Mean**	**%/SD**	**N/Mean**	**%/SD**	**N/Mean**	**P value**	**N/Mean**	**%/SD**	
General obesity status change										
$SBP_{baseline}$		103.36	11.54	106.44	13.01	108.46	11.97	111.29	12.77	<0.001
$SBP_{\text{follow-up}}$		103.49	10.84	107.21	11.56	110.58	11.31	112.69	12.32	<0.001
SBP change		0.12	12.16	0.77	13.59	2.12	12.71	1.4	13.84	<0.001
$DBP_{baseline}$		65.73	8.44	67.85	9.3	68.5	8.94	70.36	8.89	<0.001
$DBP_{\text{follow-up}}$		65.1	8.05	67.35	8.46	69.61	7.87	70.59	8.63	<0.001
DBP change		-0.63	10.07	-0.5	10.96	1.11	10.75	0.23	10.89	<0.001
$HBP_{baseline}$	no	22617	92.00%	653	82.80%	205	85.10%	2083	78.20%	<0.001
	yes	1976	8.00%	136	17.20%	36	14.90%	582	21.80%	
$HBP_{\text{follow-up}}$	no	23528	95.70%	701	88.80%	210	87.10%	2197	82.40%	<0.001
	yes	1065	4.30%	88	11.20%	31	12.90%	468	17.60%	
Abdominal obesity status change										
$SBP_{baseline}$		103.21	11.52	105.42	11.97	105.13	11.88	111.47	12.46	<0.001
$SBP_{\text{follow-up}}$		103.28	10.82	106.19	10.98	108.86	11.52	112.46	11.83	<0.001
SBP change		0.07	12.14	0.77	12.85	3.73	13.38	0.99	13.66	<0.001
$DBP_{baseline}$		65.63	8.43	67.14	8.88	67.41	8.69	70.45	8.74	<0.001
$DBP_{\text{follow-up}}$		64.96	8.02	67.12	8.17	68.33	8.61	70.41	8.48	<0.001
DBP change		-0.67	10.05	-0.02	10.8	0.92	10.53	-0.05	10.8	<0.001
$HBP_{baseline}$	no	21343	92.00%	1197	86.70%	483	89.90%	2325	78.80%	<0.001
	yes	1846	8.00%	184	13.30%	54	10.10%	627	21.20%	
$HBP_{\text{follow-up}}$	no	22198	95.70%	1253	90.70%	478	89.00%	2506	84.90%	<0.001
	yes	991	4.30%	128	9.30%	59	11.00%	446	15.10%	

SBP: systolic blood pressure. DBP: diastolic blood pressure. HBP: high blood pressure. NN: non-obese at baseline and non-obese at follow-up; NY: non-obese at baseline and obese at follow-up; YN: obese at baseline and non-obese at follow-up; YY: obese at baseline and obese at follow-up.

The adjusted means of BP change with 95% confidence interval from baseline and follow-up were presented in **Figure 1**. The relationships between changes in BP and changes in BMI or WHtR were presented in the scatter plots (**S2 Fig.**).

Association between obesity status change and risk of HBP

Association between GOB or AOB status change groups and risk of HBP in four logistic regression models were presented in **Table 3**. In the crude models, no matter for GOB or AOB, the children with continuous obese status (YY group), or newly developed obesity group (NY group), or obese before but not now (YN group) had significantly higher risks of HBP compared with the sustained non-obese children (NN group, *P*<0.001). When further adjusted for different lifestyle covariates (model 3 or model 4), the children in YN group, YY group, or NY group still had significantly higher risks of HBP compared with the sustained non-obese children (NN group, *P*<0.001). In the full adjusted models (model 4), ORs (95%CI) for the YN group, YY and NY group of GOB were 2.72 (95%CI: 2.03~3.63), 3.35 (95%CI: 1.99~5.65) and 5.03 (95%CI: 4.32~5.86), respectively. Similar results were found for the AOB status change, the ORs(95%CI) for the YN group, YY and NY group were 2.24 (95%CI: 1.76~2.85), 3.42 (95%CI: 2.36~4.95), and 4.79 (95%CI: 4.09~5.60), respectively.

~~The~~ sensitivity analyses generated similar results (**Table S2**). Further adjustment of height change or baseline HBP status did not change our results. The estimates (ORs) are very similar as before additional adjustment and still statistically significant for the NY and YY groups.

Association between risk of HBP and different quintiles of BMI or WHtR change in the obese children

For children with GOB, when categorized as quintiles by the BMI change from baseline to follow-up, only quintile 1 (BMI change ≤-0.88 kg/m^2) and quintile 2 (BMI change -0.88~ -0.21 kg/m^2) ~~is~~ significantly associated with reduced risk of HBP (OR=0.62, 95%CI: 0.46~0.84 vs OR=0.68, 95%CI: 0.50~0.91 for quintile 1 and quintile 2, respectively). A reduction of at least 0.21~0.88 kg/m^2 of BMI (only 0.86%~3.59% of the baseline BMI) in children with GOB (baseline BMI=24.49 kg/m^2) could be significantly beneficial to the BP profile compared with the reference group (**Table 4**).

For children with AOB, when categorized as quintiles by the WHtR change from baseline to follow-up, children in quintile 1 (WHtR change ≤-0.045), quintile 2 (WHtR change -0.045~ -0.024) and quintile 3(WHtR change -0.024~ -0.009) ~~group~~ were significantly associated with reduced risks of HBP (OR=0.48, 95%CI: 0.35~0.66, OR=0.69, 95%CI: 0.52~0.93 and OR=0.71, 95%CI: 0.53~0.94 for quintile 1, quintile 2 and quintile 3, respectively). A reduction of at least 0.009~0.024 (1.66%~4.42% of the baseline WHtR) of WHtR in children with AOB (baseline WHtR=0.5429) could be significantly beneficial to the BP profile (**Table 4**).

We also categorized the BMI or WHtR change as ten equal groups with percentile of P_{10}, P_{20}, P_{30}, P_{40}, P_{50}, P_{60}, P_{70}, P_{80} and P_{90} as cutoff points. Similar results were observed (**Table S3**).

Table 3. Association between risk of high blood pressure and different general of abdominal obesity status change

Obesity type	Group[a]	Model 1		Model 2		Model 3		Model 4	
		OR(95%CI)	*P*	OR(95%CI)	*P*	OR(95%CI)	*P*	OR(95%CI)	*P*
General obesity	NN	1(Ref.)		1(Ref.)		1(Ref.)		1(Ref.)	
	YN	2.77(2.20~3.49)	<0.001	2.53(2.01~3.19)	<0.001	2.53(2.00~3.20)	<0.001	2.72(2.03~3.63)	<0.001
	NY	3.26(2.23~4.78)	<0.001	3.17(2.16~4.65)	<0.001	3.15(2.13~4.66)	<0.001	3.35(1.99~5.65)	<0.001
	YY	4.71(4.19~5.29)	<0.001	4.47(3.97~5.04)	<0.001	4.79(4.23~5.43)	<0.001	5.03(4.32~5.86)	<0.001
Abdominal obesity	NN	1(Ref.)		1(Ref.)		1(Ref.)		1(Ref.)	
	YN	2.29(1.89~2.77)	<0.001	2.18(1.80~2.65)	<0.001	2.03(1.67~2.48)	<0.001	2.24(1.76~2.85)	<0.001
	NY	2.77(2.09~3.65)	<0.001	2.77(2.10~3.66)	<0.001	3.06(2.30~4.07)	<0.001	3.42(2.36~4.95)	<0.001
	YY	3.99(3.54~4.49)	<0.001	3.93(3.48~4.43)	<0.001	4.48(3.94~5.09)	<0.001	4.79(4.09~5.60)	<0.001

a: NN: non-obese at baseline and non-obese at follow-up; NY: non-obese at baseline and obese at follow-up; YN: obese at baseline and non-obese at follow-up; YY: obese at baseline and obese at follow-up. Model 1 is the crude model. Model 2 is adjusted for age and gender. Model 3 is further adjusted for province, and area. Model 4 is further adjusted for fruits consumption, vegetable consumption, sugar-sweetened beverage intake, vigorous and moderate physical activity.

Table 4. Association between risk of high blood pressure and different quintile of BMI or WHtR change in the obese

children

Variables	Group	Model 1		Model 2	
		OR(95%CI)	*P*	OR(95%CI)	*P*
BMI change (kg/m^2)	Quintile 1(≤-0.88)	0.70(0.52~0.93)	0.014	0.62(0.46~0.84)	0.002
	Quintile 2(-0.88~-0.21)	0.71(0.53~0.95)	0.020	0.68(0.50~0.91)	0.010
	Quintile 3(-0.21~0.31)	0.77(0.58~1.02)	0.073	0.77(0.58~1.03)	0.081
	Quintile 4(0.31~0.93)	1.02(0.78~1.34)	0.891	1.03(0.78~1.36)	0.828
	Quintile 5(>0.93)	1(Ref.)		1(Ref.)	
WHtR change	Quintile 1(≤-0.045)	0.64(0.49~0.85)	0.002	0.48(0.35~0.66)	<0.001
	Quintile 2(-0.045~ -0.024)	0.70(0.53~0.93)	0.012	0.69(0.52~0.93)	0.013
	Quintile 3(-0.024~ -0.009)	0.69(0.52~0.91)	0.009	0.71(0.53~0.94)	0.016
	Quintile 4(-0.009~ 0.007)	1.02(0.79~1.32)	0.888	1.03(0.79~1.33)	0.853
	Quintile 5(>0.007)	1(Ref.)		1(Ref.)	

Model 1 is the crude model. Model 2 is adjusted for age, gender, province, and area. BMI: body mass index. WHtR: waist-to-height ratio.

Discussion

In this short-term follow-up study with 28,288 children aged 6-17 years, we demonstrated the obesity status change during the short-term follow up, and compared the risk of HBP in different obesity status change groups. We found that children with persistent obesity, newly developed obesity or those converting from obese to non-obese had significantly higher risk of HBP compared with persistent non-obese children no matter for GOB or AOB. At the same time, we found that a reduction of 0.86%-3.59% of the baseline BMI in children with GOB, or reduction of 1.66%-4.42% of WHtR in children with AOB could be significantly beneficial to the BP profile.

GOB or AOB prevalence in children

During recent years, pediatric obesity has becoming an epidemic in China. Previous survey reported that the prevalence of overweight and obesity of 7-18-year-old children is 19.4% in 2014，while the corresponding prevalence ~~is~~ only 1.24% in 1985[17]. According to our study, without intervention, about 9.4% children have persistent GOB, which is more than double of the rate (3.9%) in Ziyab study among UK children[18], ~~also~~ obviously higher than Berentzen' study in Dutch[19]. Berentzen's study reported that the rate of persistent overweight in children is 4%. The difference between our study's findings with the two European studies may due to the genetic background, or the different lifestyles, or various obesogenic socio-economic environment. But obviously, this serious situation of children requires united policies, efforts from the government, school and family to tackle it.

Notably, our study identified 10.5% of the children who had persistent AOB without intervention. As far as we are concerned, no previous study reported the rate of persistent AOB with a longitudinal design among Chinese children. Undoubtedly, WHtR as an important indicator for abdominal fat accumulation, 10.5% is a high prevalence which needs united efforts to tackle this public health problem, including inter-sectional actions from government, policies in schools, and efforts from the whole families[20, 21]. Since many epidemiology studies demonstrated that AOB is closely related with risk of CVDs and diabetic related complications[22-24]. Given the big population in China, without proper interventions or control policies for pediatric obesity, it will pose a great cardiovascular disease burden related to obesity in the near future.

Different GOB or AOB status change with risk of HBP

Along with the epidemic of obesity the prevalence of HBP in children also increased in recent years, cross-sectional association between obesity and HBP has been well documented, but whether the obesity status change without intervention would have an immediate impact on HBP among children has not been concluded [25-28]. ~~Interesting,~~ our study found that those converted from obese to non-obese doubles the HBP risk compared with the persistent non-obese children, while the newly developed obesity children tripled the HBP risk of the reference group. Our finding is similar ~~with~~ a recent systematic review about weight change from childhood to adulthood and later life cardiovascular risk factors[26], showing that the related risks ~~could limited or reduced~~ when children with obesity ~~could~~ turn to be non-obese, and even eliminated when ~~the~~ obesity turn to normal weight. But at the same time there are inconsistent studies, for example, Michael’s study showed that every single period of obesity

could be detrimental to the BP profile no matter the weight status in other periods[25]. This discrepancy may due to the racial or ethnicity differences[28]. Surprisingly, in our study the newly developed obesity will sharply increase (more than triple) the risk of HBP ~~as~~ almost the same magnitude of that in persistent obese children. This is consistent with a prospective study conducted by Li et al, their study found that excessive BMI gain, especially recently BMI increase, would significantly increase BP[29].

Regarding the association between AOB status change and risk of HBP, we used WHtR cut-off point (0.5) defined AOB, which is a simple but predictive indicator often used to measure abdominal fat accumulation[12, 30, 31]. Previous longitudinal and cross-sectional studies investigated the associations between WHtR and elevated BP[24, 31-33], and found positive relation between WHtR or AOB and BP or elevated BP, but no studies investigated the longitudinal association between the change pattern of AOB status and risk of HBP. Until now, information about AOB status change in childhood is really scarce. We found the persistent or newly developed AOB can cause the same level of risk of HBP. Therefore, it is also crucial to monitor the abdominal fat accumulation, then prevent and control the development or persistence of AOB.

In addition, the sensitivity analyses generated similar results. Further adjustment of height change or baseline HBP status did not change our results. The estimates (ORs) are very similar as before additional adjustment and still statistically significant for the NY and YY groups, which shows that our findings are relative robust and stable.

Association between risk of HBP and different quintiles of BMI or

WHtR change in the obese children

~~Previous~~ study of childhood obesity intervention, 5% of the BMI reduction is significantly associated with more favorable cardiometabolic profiles (lipid, insulin sensitivity and BP)[8]. A reduction of only 0.86%-3.59% of BMI could lead to beneficial effect on HBP in our study. However, a recent meta-analysis of 54 lifestyle intervention studies in pediatric populations showed that reduction of >1 mean BMI SDS is related to improved SBP[7]. Considering the difference of race, and age in different studies, it is hard to compare or make a final quantification of the minimum favorable BMI reduction for metabolic health. But given the short-term reduction of BMI (0.86%-3.59%) or WHtR (1.66%-4.42%) among obese children could immediately have a significant effect of reducing the risk of HBP, this is encouraging for weight management in children.

Strengths and limitations

This is a large-scale study with random sampling method covering 7 provinces in China, our sample is representative and the findings could be generated to children of China. Also, we longitudinally measured the anthropometric indices and BP for all the participants, the associations between obesity status change and risk of HBP is stronger and more evident than the cross-sectional associations. Also, a variety of sensitivity analyses generated similar results, this indicates our findings is robust. For example, additional adjustment of height change (reflecting the growth effect) did not substantially change our results, suggesting that the normal growth is not likely to have considerable effect on our results. In addition, the

associations between different obesity status change and risk of HBP is verified with both GOB and AOB.

There are limitations in our study. First, our study is only a short-term follow up focused in childhood, but it is also significant because even in a short-term follow-up the newly developed obesity could be unfavorable for BP profile as persistent obesity. This means if the obesity status continues, the detrimental effects on HBP could track into later life, implying that early life intervention for the obesity is quite important for these children. Second, the BP measurement is only one-occasion measurement due to the limited founding of the study, future studies involving three-occasion BP measurement could be done to verify the findings.

Conclusion

To conclude, we examined GOB and AOB status change among Chinese children in a short-term follow-up, and the impact of the adiposity status change on BP profiles. Compared with persistent non-GOB children, children with persistent GOB had the highest risk of HBP (more than 5-fold), children with newly developed GOB had second higher HBP risk (more than triple), those converting from GOB to non-GOB has a more than double HBP risk compared with the reference group. Similar findings were also found for the relation between AOB status change with HBP risk. Reduction of only 0.86%-3.59% of BMI or only 1.66%-4.42% of WHtR among GOB or AOB children during about half a year could be significantly beneficial to the BP profile. Our results highlight the importance of preventing and controlling GOB and AOB in childhood, to reduce the cumulative detrimental effects of high BP since early life.

Acknowledgment

We would like to thank all the children for their participation in our study, also we would like to thank all the head teachers of the schools for their help and cooperation in the research. This research was funded by the National Natural Science Foundation of China (81903336, YY), the Hunan Provincial Natural Science Foundation of China (2019JJ50376, YY), Scientific Research Project of Hunan Health Committee (202112031516,YY), and a Project Supported by Scientific Research Fund of Hunan Provincial Education Department (18A0028, XY). The funders had no role in the design, analysis or writing of this article.

Funding

This research was funded by the National Natural Science Foundation of China (81903336, YY), the Hunan Provincial Natural Science Foundation of China (2019JJ50376, YY), Scientific Research Project of Hunan Health Committee (202112031516,YY), and a Project Supported by Scientific Research Fund of Hunan Provincial Education Department (18A0028, XY). The funders had no role in the study design, data collection, data analysis, writing of this article or interpreting the results.

Ethics approval and consent to participate

All procedures performed in the present study were in accordance with the Declaration of Helsinki and were approved by the Medical Ethical Committee of the Peking University Health Science Center (NO.IRB0000105213034). The author obtained permission to use the dataset from Institute of Child and Adolescent health, Peking University and the secondary analysis of the dataset without identifiable information was approved by the Ethical Committee of Hunan Normal University(NO.2019-88) and by Southern Medical

University(NO.IRB00001052-20014). Before investigation, written informed consent to participate in the study was obtained from both the children and their parents or legal guardians. The written informed consent was obtained for all forms of personally identifiable data including biomedical, clinical, and biometric data. Also, all participants had the right to withdraw from the study at any time and for any reason. All participants, together with their parents or legal guardians, had been informed that this investigation would be performed anonymously and confidentially, and their privacy would be protected and respected.

Conflict of interest

The authors declare no conflict of interest.

Availability of data and materials

The data analyzed in the present study are not publicly available due to data management rules of Peking University and Hunan Normal University, China, but are available from the corresponding author on reasonable request.

Reference

1. Dong Y, Ma J, Song Y, Ma Y, Dong B, Zou Z, et al. Secular Trends in Blood Pressure and Overweight and Obesity in Chinese Boys and Girls Aged 7 to 17 Years From 1995 to 2014. Hypertension 2018, 72(2):298-305. doi:10.1161/HYPERTENSIONAHA.118.11291.

2. GBD 2017 Risk Factor Collaborators. Global, regional, and national comparative risk assessment of 84 behavioural, environmental and occupational, and metabolic risks or clusters of risks for 195 countries and territories, 1990-2017: a systematic analysis for the Global Burden of Disease Study 2017. Lancet 2018, 392(10159):1923-1994. doi:10.1016/S0140-6736(18)32225-6

3. Dong B, Wang Z, Song Y, Wang HJ, Ma J. Understanding trends in blood pressure and their associations with body mass index in Chinese children, from 1985 to 2010: a cross-sectional observational study. BMJ Open 2015, 5(9):e009050. doi:10.1136/bmjopen-2015-009050

4. Yang Y, Dong B, Wang S, Dong Y, Zou Z, Fu L, et al. Prevalence of high blood pressure subtypes and its associations with BMI in Chinese children: a national cross-sectional survey. BMC Public Health

2017, 17(1):598. doi:10.1186/s12889-017-4522-2

5. Dong B, Ma J, Wang HJ, Wang ZQ. The association of overweight and obesity with blood pressure among Chinese children and adolescents. Biomed Environ Sci 2013, 26(6):437-444. doi:10.3967/0895-3988.2013.06.004

6. Yang Y, Lau PW, Wang J, Dong B, Wu L, Quach B, et al. Associations among cardiorespiratory endurance, body mass index and blood pressure in Han Chinese children: results from the 2010 Chinese National Survey On Students' Constitution and Health. Hypertens Res 2016, 39(11):799-804. doi:10.1038/hr.2016.63

7. El-Medany AYM, Birch L, Hunt LP, Matson RIB, Chong AHW, Beynon R, et al. What Change in Body Mass Index Is Required to Improve Cardiovascular Outcomes in Childhood and Adolescent Obesity through Lifestyle Interventions: A Meta-Regression. Child Obes 2020, 16(7):449-478. doi:10.1089/chi.2019.0286

8. Savoye M, Shaw M, Dziura J, Tamborlane WV, Rose P, Guandalini C, et al. Effects of a weight management program on body composition and metabolic parameters in overweight children: a randomized controlled trial. JAMA 2007, 297(24):2697-2704. doi:10.1001/jama.297.24.2697

9. Chen Y, Ma L, Ma Y, Wang H, Luo J, Zhang X, et al. A national school-based health lifestyles interventions among Chinese children and adolescents against obesity: rationale, design and methodology of a randomized controlled trial in China. BMC Public Health 2015, 15:210. doi: 10.1186/s12889-015-1516-9

10. National Health Commission of the People's Republic of China. Screening for overweight and obesity among school-age children and adolescents. WS/T 586—2018; 2018.

11. Schwandt P. Defining central adiposity in terms of clinical practice in children and adolescents. Int J Prev Med 2011, 2(1):1-2.

12. Liu B, Lehmler HJ, Sun Y, Xu G, Sun Q, Snetselaar LG, et al. Association of Bisphenol A and Its Substitutes, Bisphenol F and Bisphenol S, with Obesity in United States Children and Adolescents. Diabetes Metab J 2019, 43(1):59-75. doi:10.4093/dmj.2018.0045

13. National High Blood Pressure Education Program Working Group on High Blood Pressure in C, Adolescents. The fourth report on the diagnosis, evaluation, and treatment of high blood pressure in children and adolescents. Pediatrics 2004, 114(2 Suppl 4th Report):555-576.

14. Dong YH, Zou ZY, Yang ZP, Wang ZH, Jing J, Luo JY, et al. Association between high birth weight and hypertension in children and adolescents: a cross-sectional study in China. J Hum Hypertens 2017, 31(11):737-743. doi:10.1038/jhh.2017.22

15. Yang Y, Dong B, Zou Z, Wang S, Dong Y, Wang Z, et al. Association between Vegetable Consumption and Blood Pressure, Stratified by BMI, among Chinese Adolescents Aged 13-17 Years: A National Cross-Sectional Study. Nutrients 2018, 10(4):451. doi:10.3390/nu10040451

16. Gui ZH, Zhu YN, Cai L, Sun FH, Ma YH, Jing J, et al. Sugar-Sweetened Beverage Consumption and Risks of Obesity and Hypertension in Chinese Children and Adolescents: A National Cross-Sectional Analysis. Nutrients 2017, 9(12):1302. doi:10.3390/nu9121302

17. Wang S, Dong YH, Wang ZH, Zou ZY, Ma J. Trends in overweight and obesity among Chinese children of 7-18 years old during 1985-2014. Zhonghua Yu Fang Yi Xue Za Zhi 2017, 51(4):300-305. doi:10.3760/cma.j.issn.0253-9624.2017.04.005

18. Ziyab AH, Karmaus W, Kurukulaaratchy RJ, Zhang H, Arshad SH. Developmental trajectories of Body Mass Index from infancy to 18 years of age: prenatal determinants and health consequences. J Epidemiol Community Health 2014, 68(10):934-941. doi:10.1136/jech-2014-203808

19. Berentzen NE, van Rossem L, Gehring U, Koppelman GH, Postma DS, de Jongste JC, et al. Overweight patterns throughout childhood and cardiometabolic markers in early adolescence. Int J Obes (Lond) 2016, 40(1):58-64. doi:10.1038/ijo.2015.196

20. Jia P, Li M, Xue H, Lu L, Xu F, Wang Y. School environment and policies, child eating behavior and overweight/obesity in urban China: the childhood obesity study in China megacities. Int J Obes (Lond) 2017, 41(5):813-819. doi:10.1038/ijo.2017.2

21. Seidell JC, Halberstadt J. The global burden of obesity and the challenges of prevention. Ann Nutr Metab 2015, 66 Suppl 2:7-12. doi:10.1159/000375143

22. Wan H, Wang Y, Xiang Q, Fang S, Chen Y, Chen C, et al. Associations between abdominal obesity indices and diabetic complications: Chinese visceral adiposity index and neck circumference. Cardiovasc Diabetol 2020, 19(1):118. doi:10.1186/s12933-020-01095-4

23. Teixeira FDC, Pereira FEF, Pereira AF, Ribeiro BG. Overweight or obesity and abdominal obesity and their association with cardiometabolic risk factors in Brazilian schoolchildren: A cross-sectional study. Nutrition 2020, 78:110780. doi:10.1016/j.nut.2020.110780

24. Masocha V, Monyeki MA, Czyz SH. Longitudinal relationships between changes in body composition and changes in selected metabolic risk factors (abdominal obesity and blood pressure) among South African adolescents. PeerJ 2020, 8:e9331. doi:10.7717/peerj.9331

25. Merten MJ.Weight status continuity and change from adolescence to young adulthood: examining disease and health risk conditions. Obesity (Silver Spring) 2010, 18(7):1423-1428. doi:10.1038/oby.2009.365

26. Sun J, Xi B, Yang L, Zhao M, Juonala M, Magnussen CG. Weight change from childhood to adulthood and cardiovascular risk factors and outcomes in adulthood: A systematic review of the literature. Obes Rev 2020: (Online ahead of print). doi:10.1111/obr.13138

27. Gooding HC, de Ferranti SD. Cardiovascular risk assessment and cholesterol management in adolescents: getting to the heart of the matter. Curr Opin Pediatr 2010, 22(4):398-404. doi: 10.1097/MOP.0b013e32833a6e22

28. Suglia SF, Clark CJ, Gary-Webb TL. Adolescent obesity, change in weight status, and hypertension: racial/ethnic variations. Hypertension 2013, 61(2):290-295. doi: 10.1161/HYPERTENSIONAHA.111.00214

29. Li L, Law C, Power C. Body mass index throughout the life-course and blood pressure in mid-adult life: a birth cohort study. J Hypertens 2007, 25(6):1215-1223.10.1097/HJH.0b013e3280f3c01a

30. Ashwell M, Gibson S. Waist-to-height ratio as an indicator of 'early health risk': simpler and more predictive than using a 'matrix' based on BMI and waist circumference. BMJ Open 2016, 6(3):e010159. doi:10.1136/bmjopen-2015-010159

31. Graves L, Garnett SP, Cowell CT, Baur LA, Ness A, Sattar N, Lawlor DA. Waist-to-height ratio and cardiometabolic risk factors in adolescence: findings from a prospective birth cohort. Pediatr Obes 2014, 9(5):327-338. doi: 10.1111/j.2047-6310.2013.00192.x

32. Zhang YX, Wang ZX, Chu ZH, Zhao JS. Blood pressure profiles of children and adolescents categorized by waist-to-height ratio cutoffs: study in a large sample in Shandong, China. Blood Press Monit 2017, 22(3):143-148. doi: 10.1097/MBP.0000000000000249

33. Choi JR, Koh SB, Choi E. Waist-to-height ratio index for predicting incidences of hypertension: the ARIRANG study. BMC Public Health 2018, 18(1):767. doi: 10.1186/s12889-018-5662-8

Figure Legend

Figure 1. Adjusted means and 95% confidence interval of blood pressure change from baseline to the follow-up in different general or abdominal obesity status change groups. NN: non-obese at baseline and non-obese at follow-up; NY: non-obese at baseline and obese at follow-up; YN: obese at baseline and non-obese at follow-up; YY: obese at baseline and obese at follow-up. Means were adjusted for age, gender, area, BMI, fruit consumption, vegetable consumption, sugar-sweetened beverage intake and physical activities.

Supplementary information

S1 Table. Body Mass Index Reference Norm for Screening Overweight and Obesity Among Chinese children aged 6-18 years (kg/m^2).

S2 Table. Association between risk of HBP and different obesity status change with

adjustment of other potential covariates.

S3 Table. Association between risk of high blood pressure and different groups (ten equal groups) of BMI or WHtR change in the obese children.

S1 Fig. The size of one serving of fruit or vegetable (as the size of a fist of ordinary adult).

S2 Fig. The relationships between changes in blood pressures and changes in body mass index (BMI) or waist-to-height ratio (WHtR).

S1 File. Questionnaire about dietary factors and physical activities in children and adolescents(including S1 Fig.)

Figure
Click here to access/download;Figure;Figure 1.pptx

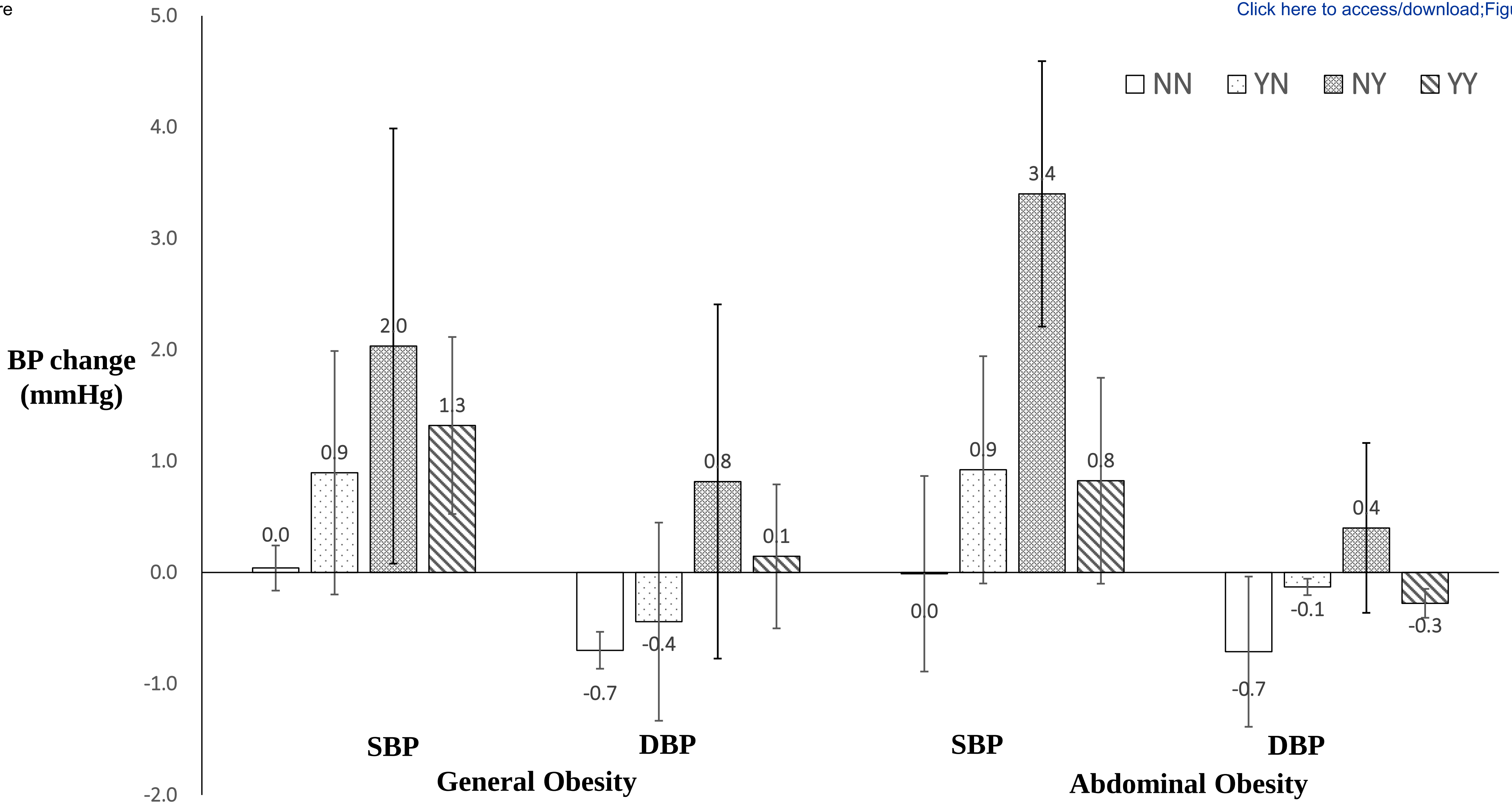

Figure 1. Adjusted means and 95% confidence interval of blood pressure change from baseline to the follow-up in different general or abdominal obesity status change groups.

Click here to access/download
Supporting Information
Supplementary information.pdf
